# Fabrication of CdTe QDs/BiOI-Promoted TiO_2_ Hollow Microspheres with Superior Photocatalytic Performance Under Simulated Sunlight

**DOI:** 10.1186/s11671-019-2878-1

**Published:** 2019-02-06

**Authors:** Xiaofei Qu, Meihua Liu, Longfei Li, Chunqi Wang, Cuihua Zeng, Jianhuang Liu, Liang Shi, Fanglin Du

**Affiliations:** 1 College of Materials Science and Engineering, Qingdao University of Science and Technology, Zhengzhou Road 53, Qingdao, 266042 China; 2 Ansteel Cold Rolling (PuTian) Co., Ltd., Wangshan East Road 555, Putian, 351100 China

**Keywords:** Photocatalysis, Ternary heterojunctions, CdTe quantum dots, TiO_2_ hollow microspheres

## Abstract

Hollow and heterostructured architectures are recognized as an effective approach to improve photocatalytic performance. In this work, ternary TiO_2_/CdTe/BiOI with hollow structure was constructed via a step-by-step method. In addition, the effect of TiO_2_ structural regulation and the energy band alignment of BiOI and CdTe quantum dots (CdTe QDs) with TiO_2_ in TiO_2_/CdTe/BiOI on photocatalytic dye removal were also studied. The results reveal that the TiO_2_/CdTe/BiOI heterostructures with hollow substrates exhibit much higher photocatalytic activities than pure TiO_2_, P25, TiO_2_/CdTe, and TiO_2_/BiOI and ternary TiO_2_/CdTe/BiOI with solid substrates. For TiO_2_(H)/CdTe/BiOI, several synergistic factors may be responsible for the remarkable visible-light photodegradation performance, such as strong visible-light absorption by BiOI and larger specific surface area.

## Background

Owing to the energy saving and environmentally friendly merits, semiconductor photocatalysis has drawn increasing interests in environmental conservation. Photocatalysts can be used in various aspects, such as self-cleaning, water treatment, air purification, and anti-bacteria [1, 2]. Among them, due to the advantages of low cost, excellent stability, and nontoxicity [3], titanium dioxide (TiO_2_) has been extensively investigated. However, it can only utilize small portion of solar spectrum because of its wide bandgap and relatively rapid charge recombination, limiting the photo-conversion efficiency [4].

In order to improve the visible-light photocatalytic efficiency of titania, various strategies have been adopted including ion doping, noble metal loading, heterojunction constructing, and sensitization [5–8]. Among these strategies, heterojunction formed by coupling with narrow bandgap semiconductor is supposed to be one of the most effective methods to improve the visible-light response and reduce charge recombination simultaneously [9].

Bismuth oxyhalides have attracted considerable attention due to low cost, good stability, and wide light-response range [10, 11], which have an isotropic-layered structure with [Bi_2_O_2_]^2+^ layers intercalated by X^−^ ions (X = F, Cl, Br, I) [12]. Among the bismuth oxyhalides, BiOI with the smallest bandgap (1.72–1.9 eV) [13] has been proven to be an efficient visible-light photocatalyst for the degradation of RhB [14] and MO [15]. The internal electric field between the [Bi_2_O_2_]^2+^ and I^−^ layers can promote the separation of photo-induced charges and enhance the photocatalytic activity [16]. Although both the conduction band and valence band potentials lie between those of TiO_2_, type II heterojunction could be formed by coupling p-type BiOI and n-type TiO_2_ together when Fermi levels reach equilibrium, thus making conduction band electrons of BiOI migrate to TiO_2_ [17]. Up to now, although many efforts have been devoted to develop binary heterostructured photocatalysts, the limited visible-light response and relatively low charge separation efficiency are still the stumbling block.

To solve the issues mentioned above, multi-component heterojunction systems have been developed. Cadmium telluride (CdTe), as an important p-type II−VI compound semiconductor, has received much attention because of its direct bandgap of 1.44 eV [18] and a large optical absorption coefficient in the solar spectrum [19]. CdTe quantum dots (CdTe QDs) have been widely used to modify various semiconductors: Feng et al. [20] synthesized the CdTe-decorated TiO_2_ nanotube arrays via a pulse electrodeposition method, and the results indicated CdTe/TiO_2_ nanotube arrays (CdTe/TiO_2_ NTAs) exhibited outstanding photocatalytic property than the bare TiO_2_ NTAs; Liu et al. [21] reported the synthesis of CdTe/ZnO nanocomposites by a hot bath method, and the results showed that CdTe/ZnO owed a better photocatalytic activity for Rhodamine B than bare ZnO. However, apart from binary heterostructured photocatalysts, ternary-heterostructured TiO_2_/BiOI modified with CdTe QDs may present fascinating photocatalytic performance and be worthy of being studied further.

In the present work, CdTe QDs/BiOI-modified TiO_2_ was prepared for the photocatalytic application by a two-step method, and solid and hollow TiO_2_ microspheres were applied as precursors. In addition, the structural evolution of TiO_2_/CdTe/BiOI composites and the synergetic effect of CdTe and BiOI in photocatalytic process were also studied in detail.

## Methods

### Materials

Titanium isopropoxide (TTIP, 97%) and bismuth nitrate pentahydrate (AR, 99.0%) were purchased from Macklin Inc. Cadmium chloride hemi-pentahydrate (CdCl_2_·2.5H_2_O, 99.0%), sodium tellurite (Na_2_TeO_3_, 98.0%), *N*-acetyl-*L*-cysteine (98.0%), potassium borohydride (KBH_4_, 97%), sodium hydroxide (NaOH, 96.0%), potassium nitrate (KNO_3_, 99%), potassium bromide (KBr, 99%), hydrochloric acid (HCl, 36–38%), hydrogen peroxide (H_2_O_2_, 30%), ethylene glycol, and absolute ethanol were all analytical grade and purchased from Sinopharm Chemical Reagent Co., Ltd.

### Synthesis of TiO_2_ Solid Microspheres and Hollow Microspheres

In a typical procedure, 0.8 mL of KNO_3_ solution (0.1 mol/L) was dissolved into 200 mL of ethanol. Then, 4.4 mL of TTIP was added to the above solution and stirred until white precipitate  was generated. Aged for 12 h, the obtained white suspension was centrifuged and washed with deionized water and ethanol for several times, and the amorphous TiO_2_ (TiO_2_·nH_2_O) could be obtained. After being further dried at 60 °C for 12 h and calcined at 450 °C for 2 h, TiO_2_ solid microspheres (TiO_2_(S)) could be gained.

As for TiO_2_ hollow microspheres, they were fabricated via a hydrothermal process. Typically, TiO_2_·nH_2_O (200 mg) were dispersed into 40 mL of 0.05 wt% H_2_O_2_ and stirred for 10 min. Then, 480 mg of NaOH powders was dissolved into the suspension above, and the mixture was transferred into a Teflon-lined autoclave and was kept at 180 °C for 4 h. The precipitates were collected and then were immersed in hydrochloride acid (0.1 mol/L). After being washed with deionized water several times, the resultants were dried and calcined as the previous procedure, and thus, TiO_2_ hollow microspheres, labeled as TiO_2_(H), were obtained.

### Synthesis of TiO_2_ Spheres Modified with CdTe QDs

TiO_2_ powders (2.0 g) were dispersed in 40 mL of deionized water, and then, 97.9 mg of *N*-acetyl-*L*-cysteine, 114.2 mg of CdCl_2_·2.5H_2_O, and 178 mg of KBH_4_ were subsequently added into the mixture at 30 min intervals. Afterwards, 10 mL Na_2_TeO_3_ (0.01 mol/L) aqueous solution was added into the above mixture with 5 mL/min. Then, the temperature of the system was elevated to 100 °C in 30 min and refluxed for 6 h. Finally, the products were washed with water and ethyl alcohol several times and dried at 60 °C for 12 h, and the products were labeled as TiO_2_/CdTe*.*

### Fabrication of TiO_2_/CdTe/BiOI Ternary Composites

Briefly, TiO_2_/CdTe powders (258 mg) obtained above were dispersed in 10 mL of EG, forming a white suspension. Afterwards, a Bi(NO_3_)_3_ solution prepared by dissolving 627.6 mg of Bi(NO_3_)_3_ in 28 mL of EG was dropped into the above suspension in 15 min. Then, a solution containing 214.8 mg of KI and 24 mL of EG was dropped into the previous mixture. After stirring for 1 h, a yellow solution was transferred into the Teflon-lined autoclave and was kept at 80 °C for 3 h. The resulting precipitates were collected, sufficiently washed with ethanol and deionized water, and dried, which were labeled as TiO_2_/CdTe/BiOI.

Binary TiO_2_/BiOI heterostructures were obtained via the similar procedure by adding single component TiO_2_ in the solution above.

### Characterization

X-ray diffraction (XRD) patterns were obtained on a Rigaku D-MAX2500 X-ray diffractometer fitted with Cu Kα radiation. Scanning electron microscopy (SEM) was obtained using JSM-6700F field emission scanning electron microscope (JEOL, Japan). Transmission electron microscopy (TEM) images of the samples were obtained on a JEM-2100 microscope (JEOL, Japan) at an accelerating voltage of 200 kV. Nitrogen adsorption/desorption isotherms were measured at 77 K using a surface area and pore size analyzer (NOVA 1000e, Quantachrome Instruments), and the Brunauer-Emmett-Teller (BET) method was applied to evaluate the specific surface area. X-ray photoelectron spectroscopy (XPS) analysis was performed on an XSAM800 (Kratos Corporation, UK) with an Al Kα (1486.6 eV) achromatic X-ray source. The optical absorption spectra and diffuse reflection spectra (DRS) were obtained using an ultraviolet-visible spectrophotometer (CARY500UV, Varian). Photoluminescence spectra were recorded using Shimadzu RF-5301 with an excitation wavelength of 365 nm. Transient photocurrent was analyzed by an electrochemical workstation (CHI760E, Shanghai Chenhua, China) with a standard three-electrode system which used catalyst-deposited FTO glass as working electrode, Pt as the counter electrode, and SCE as the reference electrode in the electrolyte of 0.5 M Na_2_SO_4_.

### Photocatalytic Performance

Photocatalytic activity of the synthesized composites was tested based on the degradation of methyl orange (MO), using 500 W Xe-arc lamp as light source. Here, an amount of 140 mg of photocatalysts was added to 50 mL of MO solution (5 mg/L) taken in a quartz beaker. Prior to illumination, the suspension was stirred for 30 min in a dark chamber to achieve adsorption-desorption equilibrium between photocatalysts and MO solution. The mixture was then irradiated for 180 min, and 4 mL aliquot of solution was sampled at every 45 min intervals. The mixture was centrifuged at 8000 rpm for 3 min in order to remove the catalysts from the supernatant. In the stability test of photocatalyst, the samples were washed with deionized water after each cycle and then added to a fresh MO solution for the next cycle. The residual concentration of MO was monitored using a UV-vis spectrophotometer (CARY500UV, Varian), and the degradation percentage was quantitatively analyzed by comparing the maximum absorption at 465 nm.

Generally, holes (h^+^), electrons (e^−^), superoxide radicals (•O^−^_2_), and hydroxyl radicals (•OH) could be produced in the semiconductor photocatalytic system under irradiation. A radical scavenging test for the photo-induced active species was carried out. The scavenging experiment was similar to the photocatalytic decomposition tests, where ethylenediaminetetraacetic acid disodium salt (EDTA-2Na, 2 mmol/L), potassium bromate (KBrO_3_, 10 mmol/L), benzoquinone (BQ, 1 mmol/L), and isopropyl alcohol (IPA, 10 mmol/L) were used for scavenging the h^+^, e^−^, •O^−^_2_, and •OH reactive species, respectively.

## Results and Discussions

**Fig. 1:**
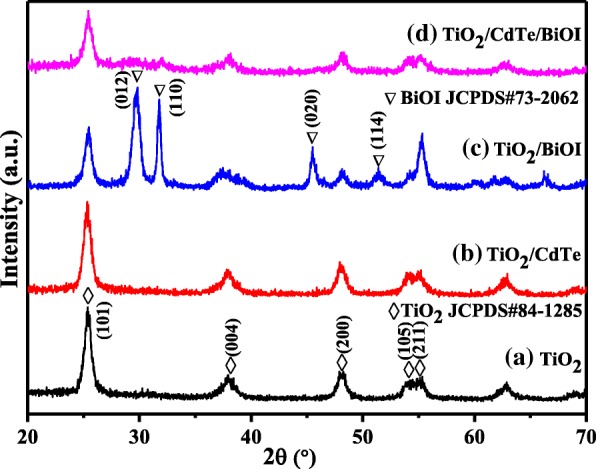
XRD patterns of **a** bare TiO_2_, **b** TiO_2_/CdTe, **c** TiO_2_/BiOI, and **d** TiO_2_/CdTe/BiOI

Figure 1 presents the powder X-ray diffraction patterns of TiO_2_, TiO_2_/CdTe, TiO_2_/BiOI, and TiO_2_/CdTe/BiOI. It is noted that TiO_2_ characteristic peaks could be picked out in all the samples, and the peaks with 2θ values at 25.5°, 37.8°, 48.8°, 53.5°, and 55.6° can be indexed to (101), (004), (200) and (105), (211) facets of anatase (JCPDS #84-1285) [22]. From the patterns shown in Fig. 1c, d, the diffraction peaks at 2θ of 29.7°, 31.7°, 45.5°, and 51.3° are exhibited besides the peaks of TiO_2_, which is consistent with tetragonal BiOI (JCPDS #73-2062) [23]. However, the peaks of BiOI are much more evident in TiO_2_/BiOI than those of TiO_2_/CdTe/BiOI. As for TiO_2_/CdTe (Fig. 1b), no other peaks are found clearly in the XRD pattern besides TiO_2_, because of the small crystallite size or a tiny amount dosage of CdTe QDs.

**Fig. 2:**
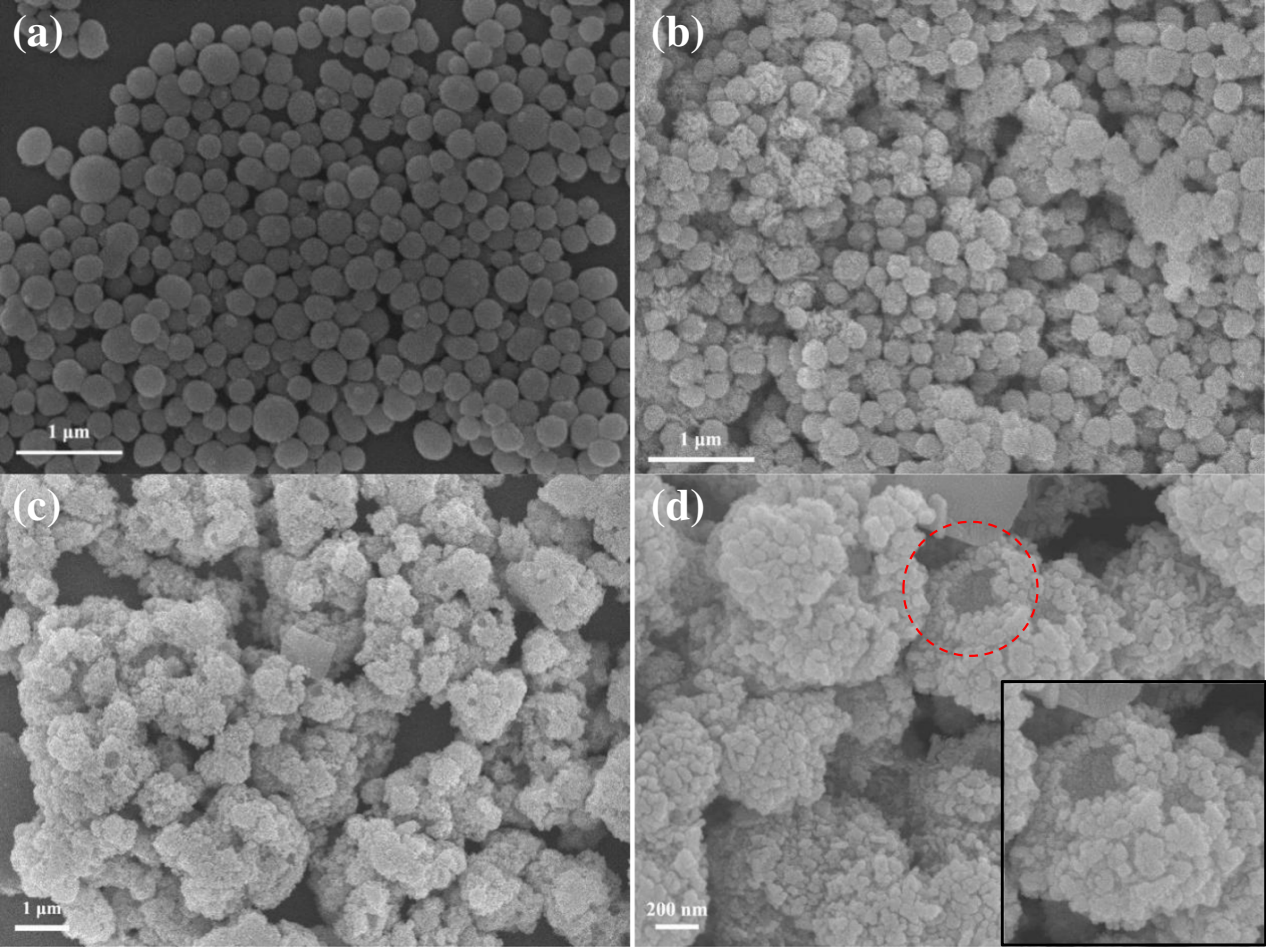
SEM images of **a** TiO_2_(S)/CdTe, **b** TiO_2_(S)/CdTe/BiOI, and **c**–**d** TiO_2_(H)/CdTe/BiOI

The external morphology of binary TiO_2_/CdTe and ternary TiO_2_/CdTe/BiOI is characterized using SEM, as displayed in Fig. 2. The overall observation of all the TiO_2_-based composites exhibits a spherical morphology, with a diameter about 200-400 nm, and flake-like BiOI deposited on the surface of TiO_2_ spheres except TiO_2_/CdTe shown in Fig. 2a. The SEM image of TiO_2_(S)/CdTe/BiOI displayed in Fig. 2b shows a similar situation as TiO_2_(S)/CdTe, and the TiO_2_(S) spheres are covered by plenty of BiOI flakes with relatively regular morphologies. However, the case is quite different for TiO_2_(H)/CdTe/BiOI, in which the particles possess nonuniform morphology with various size and cauliflower-like surface, as shown in Fig. 2c, d. Moreover, the BiOI flakes seem to be easier to attach on TiO_2_(H)/CdTe caused by rough surface originated from TiO_2_(H), and the pristine hollow structure of TiO_2_(H) matrix could be identified in the magnified view.

**Fig. 3:**
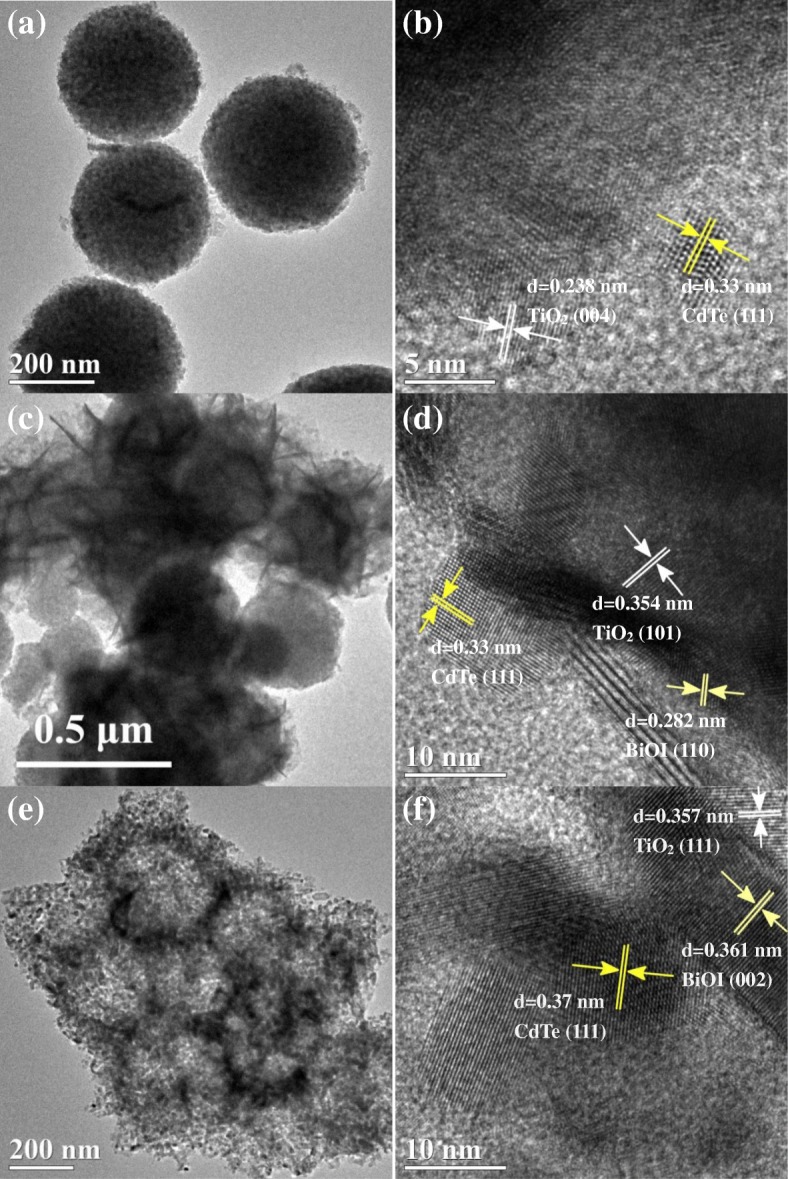
TEM images of **a**–**b** TiO_2_(S)/CdTe, **c**–**d** TiO_2_(S)/CdTe/BiOI, and **e**–**f** TiO_2_(H)/CdTe/BiOI

TEM images give further insight into the structural evolution of the ternary TiO_2_/CdTe/BiOI, as shown in Fig. 3. Uniform-sized and well-dispersed solid spherical TiO_2_ can be clearly observed in Fig. 3a, and some tiny particles are also found in the region. Lattice spacing of 0.238 nm and 0.33 nm corresponding to the (004) plane of TiO_2_ and the (111) plane of CdTe can be identified in Fig. 3b, demonstrating formation of heterojunctions between TiO_2_ spheres and CdTe QDs. It can be seen from Fig. 3c that numerous flakes were attached to the surface of solid spherical TiO_2_, and the lattice fringe was 0.282 nm in Fig. 3d, which is in accordance with the [110] direction of BiOI crystal. Moreover, the magnification of the interface also testifies the existence of CdTe QDs and BiOI flakes in TiO_2_(S)/CdTe/BiOI. In contrast, Fig. 3e exhibits the TiO_2_(H)/CdTe/BiOI which appears in the morphology of aggregated hollow spheres without apparent flake-like grains attached. The phenomenon implies BiOI components probably consist in much more smaller particles as the huge surface of TiO_2_(H) spheres, which is composed by plenty of primary TiO_2_ nanoparticles, decentralizes the nucleation sites. Similarly, the magnified view of the interface confirms the presence of heterostructured ternary TiO_2_(H)/CdTe/BiOI composites, as shown in Fig. 3f, and the (111) crystal plane of CdTe and (002) of BiOI can be found clearly.

**Fig. 4:**
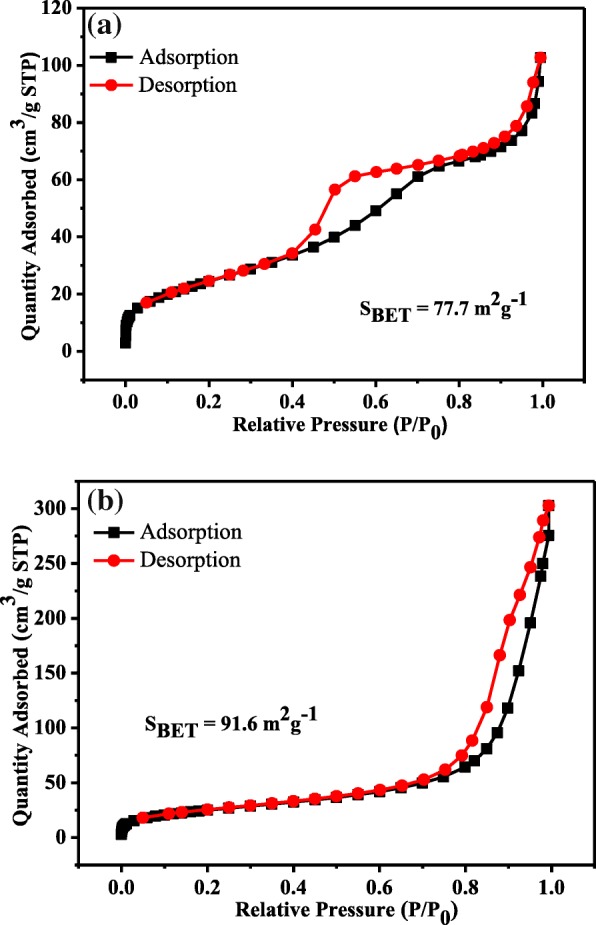
Nitrogen absorption-desorption isotherms of **a** TiO_2_(S)/CdTe/BiOI and **b** TiO_2_(H)/CdTe/BiOI

Figure 4 shows the nitrogen adsorption/desorption isotherms of the ternary TiO_2_(S)/CdTe/BiOI and TiO_2_(H)/CdTe/BiOI heterostructures. Both the isotherms exhibit similar characteristics of a type IV isotherm, according to the classification of IUPAC [24]. However, the TiO_2_(S)/CdTe/BiOI possesses a H2 type hysteresis loop in the range 0.4-0.8 [25], which indicates the accumulation of uniform TiO_2_ grains by direct calcination, while TiO_2_(H)/CdTe/BiOI with a H3 type hysteresis loop due to staking pores is derived from hydrothermal nanosheets or nanotube precursors [10], and the results are in accordance with SEM and TEM images. In addition, the specific surface area of TiO_2_(S)/CdTe/BiOI and TiO_2_(H)/CdTe/BiOI was calculated to be 77.7 and 91.6 m^2^ g^−1^ using the Brunauer-Emmett-Teller (BET) method. A relatively larger surface area of TiO_2_(H)/CdTe/BiOI microspheres could provide more active sites for the adsorption of reactant molecules, resulting a more efficient photocatalytic performance.

**Scheme 1:**
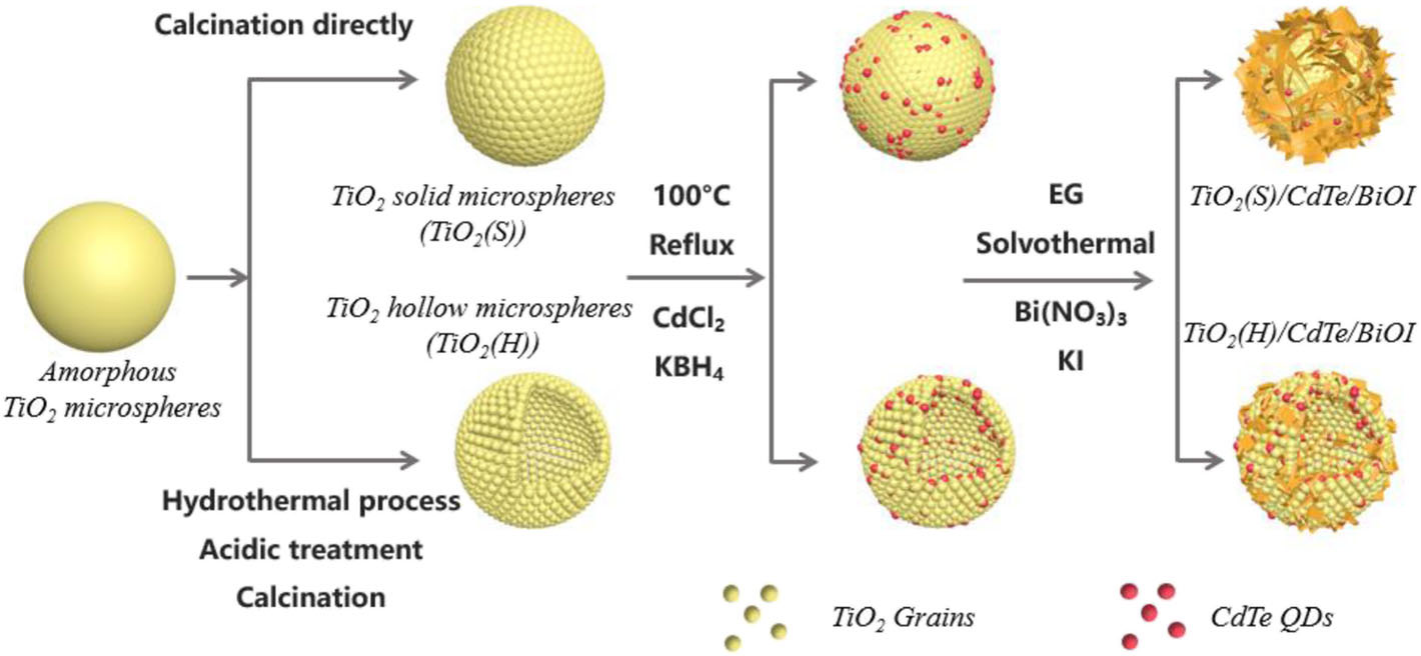
Structural evolution from amorphous TiO_2_ to TiO_2_/CdTe/BiOI heterostructures

According to the discussion above, the evolution of TiO_2_/CdTe/BiOI heterostructures can be illustrated by Scheme 1. Alternatively, amorphous TiO_2_ microspheres can also transform into TiO_2_ solid microspheres directly via a simple calcination procedure. During the following hydrothermal process, the pre-existing TiO_2_/CdTe QD microspheres could act as nucleation sites for BiOI crystallization, and thus, the ternary TiO_2_/CdTe/BiOI heterostructures form simultaneously. It is well known that BiOI has an intrinsic rapid nucleation rate and growth rate; as a result, BiOI particles with relatively large size and few quantity form on the TiO_2_(S), in a flake-like shape as growing along the [110] direction. However, in the case of TiO_2_(H) microspheres, which possess larger surface for BiOI crystallization, BiOI particles tend to exist in a form which is relatively small in size and abundant in quantity. Therefore, the surface morphology of TiO_2_ microspheres determines the shape and form of BiOI particles, as the SEM and TEM results suggested.

**Fig. 5:**
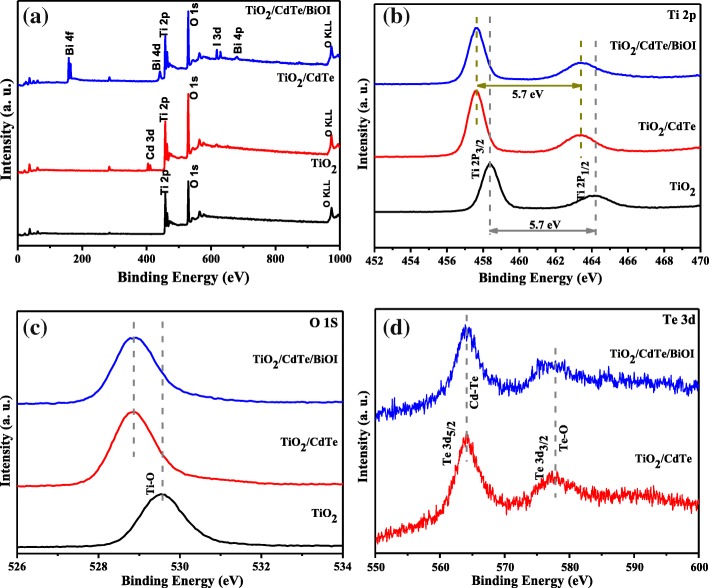
**a** XPS scanning spectrum of the samples of TiO_2_, TiO_2_/CdTe, TiO_2_/CdTe/BiOI. **b**–**d** High-resolution XPS spectra of Ti, O, and Te

XPS is employed to investigate the surface electronic states and the chemical composition of the as-prepared samples, and the results are shown in Fig. 5. It can be seen from the XPS survey spectrum that the TiO_2_/CdTe/BiOI contains Ti, O, Bi, I, Cd, and Te elements. Figure 5b shows that the peaks in the high-resolution XPS spectrum of Ti 2p corresponding to binding energies of 458.5 eV and 464.2 eV are attributed to Ti 2p_3/2_ and Ti 2p_1/2_, which indicated a Ti(IV) oxidation state [26], and the measured binding energy (BE) separation of 5.7 eV is consistent with TiO_2_ [27]. However, the binding energies of Ti 2p_1/2_ and Ti 2p_3/2_ for TiO_2_/CdTe and TiO_2_/CdTe/BiOI are 457.5 and 463.3 eV, respectively, both of which have a shift about 0.8 eV to the lower energy region compared with bare TiO_2_, further proving a strong interaction between CdTe and TiO_2_. The O 1 s core level spectrum (Fig. 5c) at around 529.5 eV is associated with the lattice O in the bare TiO_2_ samples, which is related to Ti-O bonds [28]. It deserves to be mentioned that a shift towards the lower binding energy of the O 1 s peak in the TiO_2_/CdTe and TiO_2_/CdTe/BiOI samples indicates loss of oxygen ions [27], which can be attributed to the partial oxidation of CdTe on TiO_2_. This result fits well with the shift of Ti 2p and implies the formation of the interface among TiO_2_, BiOI, and CdTe in heterojunction. The Te 3d_5/2_ spectra (Fig. 5e) reveal two states of tellurium at BE of 564.1 eV and 577.1 eV, respectively, which are characteristic for CdTe.

**Fig. 6:**
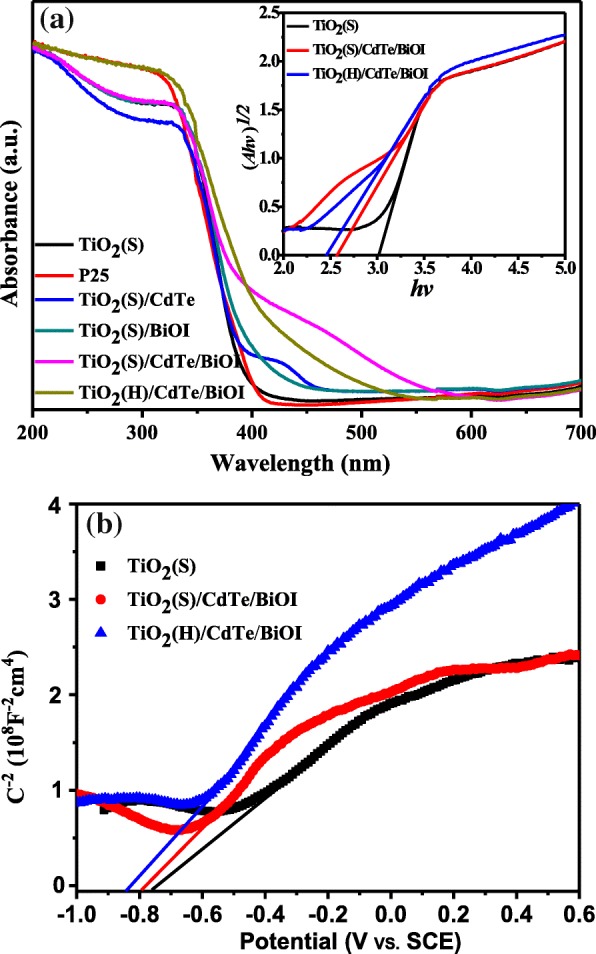
**a** UV-Vis diffuse-reflectance spectra of all samples and corresponding Tauc plots of TiO_2_(S), TiO_2_(S)/CdTe/BiOI, and TiO_2_(H)/CdTe/BiOI samples. **b** Mott-Schottky plots in 0.5 M Na_2_SO_4_ at a frequency of 1 KHz of TiO_2_(S), TiO_2_(S)/CdTe/BiOI, and TiO_2_(H)/CdTe/BiOI samples

As the optical adsorption properties are supposed critical for the multi-component semiconductors, the optical absorption spectra of TiO_2_(S), TiO_2_(S)/CdTe, TiO_2_(S)/BiOI, TiO_2_(S)/CdTe/BiOI, and TiO_2_(H)/CdTe/BiOI are shown in Fig. 6a. The as-prepared TiO_2_ shows a similar trend as commercial P25, suggesting a typical ultraviolet response characteristic. As for the binary and ternary composites, the TiO_2_(S)/CdTe/BiOI possesses a much more obvious absorption in the visible region than TiO_2_(S)/CdTe and TiO_2_(S)/BiOI, reflecting an enhanced connection among the components by CdTe QD loading. And the absorption of TiO_2_(S)/CdTe/BiOI in the visible range is a little weaker than that of TiO_2_(H)/CdTe/BiOI; we attribute this phenomenon to the dispersive effect by hollow-structured TiO_2_ microspheres as discussed above. The insert image shows the Tauc plots of *(Ahv)*^*1/2*^ versus the *hv* of samples. The bandgaps of TiO_2_(S), TiO_2_(S)/CdTe/BiOI, and TiO_2_(H)/CdTe/BiOI are estimated by extrapolating the straight line to the abscissa axis and estimated to be 3.02 eV, 2.57 eV, and 2.45 eV, respectively. It is worth noting that TiO_2_(S)/CdTe/BiOI displays a small bulge different from TiO_2_(H)/CdTe/BiOI, regardless of the blue shift by 0.12 eV, suggesting nonuniform distribution of large BiOI particles as SEM results implied above.

In order to further comprehend the electronic properties and structure of both photocatalysts, the electrochemical Mott-Schottky experiment is performed in 0.5 M Na_2_SO_4_, as shown in Fig. 6b. From Fig. 6b, all samples show a positive slope in the Mott-Schottky plots, indicating that prepared composites are apparent n-type semiconductors [29, 30]. And the conduction band position (CB) energy of samples can be approximately equal to the flat-band potential (*E*_fb_) via extrapolating the *X* intercepts of the linear portion in the Mott-Schottky plots. The value of *E*_fb_ of the TiO_2_(S), TiO_2_(S)/CdTe/BiOI, and TiO_2_(H)/CdTe/BiOI is found to be − 0.76 V (vs SCE), − 0.80 V (vs SCE), and − 0.85 V (vs SCE), respectively. Since the SCE we used in the Mott-Schottky measurement possesses a value of − 0.24 V versus NHE [31], the CBM of TiO_2_(S), TiO_2_(S)/CdTe/BiOI, and TiO_2_(H)/CdTe/BiOI samples could be calculated to be − 0.52 V (vs NHE), − 0.56 V (vs NHE), and − 0.6 V (vs NHE), respectively, which is more negative than the reduction potential of O_2_/•O^−^_2_ (E^0^(O_2_/•O^−^_2_) = − 0.33 V vs NHE). Furthermore, combined with the bandgap value from Fig. 6, the valence band maximum (VBM) is located at 2.5 V (vs NHE), 2.01 V (vs NHE), and 1.85 V (vs NHE), respectively.

**Fig. 7:**
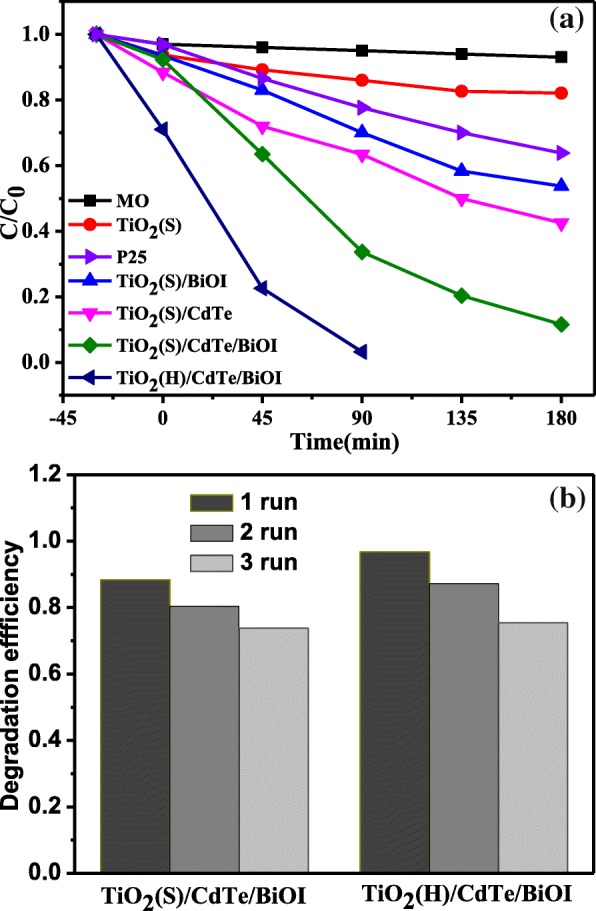
**a** Photocatalytic performance of the samples for MO removal. **b** Photocatalytic recycle experiments for the TiO_2_(S)/CdTe/BiOI and TiO_2_(H)/CdTe/BiOI samples

Figure 7a shows the photocatalytic degradation of MO in solutions without catalyst and over different photocatalysts. It can be clearly seen that TiO_2_ solid microspheres and P25 possess relatively poor photocatalytic activities under simulated sunlight, and the degradation is caused by the small ultraviolet portion from the light source. In contrast, the photocatalytic performance of TiO_2_(S)/BiOI and TiO_2_(S)/CdTe is slightly enhanced, and the MO removal percentage reached 46.3% and 57.5% after 180 min irradiation, respectively. It is worth noting that the photocatalytic degradation of MO could achieve 88.4% in 180 min and 99.7% in 90 min for TiO_2_(S)/CdTe/BiOI and TiO_2_(H)/CdTe/BiOI, respectively, due to synergistic binary visible-responding component BiOI and CdTe QDs. Furthermore, the more efficient TiO_2_(H)/CdTe/BiOI over TiO_2_(S)/CdTe/BiOI is probably caused by a slightly larger specific surface area (91.6 m^2^ g^−1^ over 77.7 m^2^g^−1^) as mentioned above. Figure 7b shows the cycle degradation experiment of TiO_2_(S)/CdTe/BiOI and TiO_2_(H)/CdTe/BiOI composites. There is a slight reduce of the photodegradation efficiency after three cycles.

**Fig. 8:**
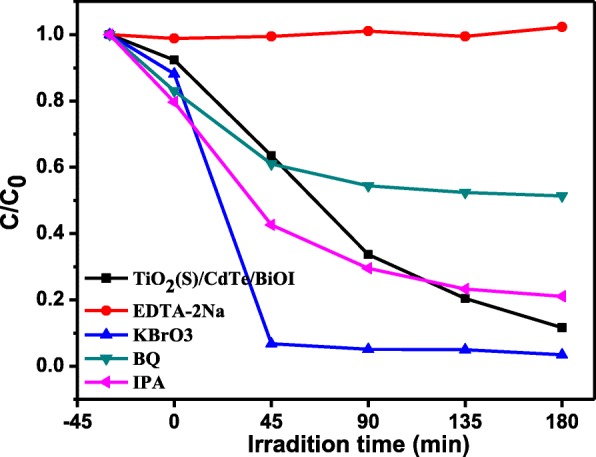
The plots of photodegraded performance of MO under trapping the different photogenerated active species of TiO_2_(S)/CdTe/BiOI sample

In order to evaluate the pathway of the MO photocatalytic degradation by the TiO_2_/CdTe/BiOI, the effect of h^+^, e^−^, •OH, and •O^−^_2_ was investigated by trapping experiment using EDTA-2Na (h^+^), KBrO_3_ (e^−^), BQ (•O^−^_2_), and IPA (•OH). Figure 8 shows the degradation efficiency of MO during these photocatalytic experiments in the presence of the selected scavengers. It can be found that the photocatalytic process is suppressed than without any scavenger and the degradation efficiency is almost none in the presence of EDTA-2Na. However, the e^−^ scavenger could accelerate the degradation, which demonstrated that the holes (h^+^) are the main active species for the MO degradation. The BQ as •O^−^_2_ scavenger just influenced the degradation in a small degree, suggesting that •O^−^_2_ are partially responsible for the photocatalytic oxidation process. In addition, the influence of IPA on the TiO_2_/CdTe/BiOI sample removing MO is hardly observed, indicating that •OH radicals are hardly useful in the current photocatalytic system.

**Fig. 9:**
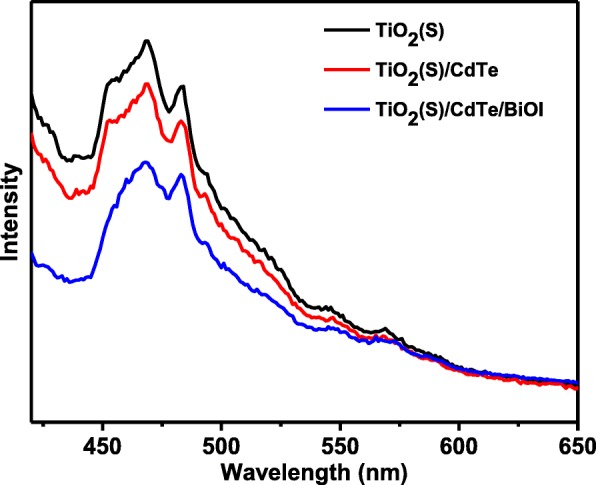
Photoluminescence spectra of the bare TiO_2_(S), TiO_2_(S)/CdTe/BiOI, and TiO_2_(H)/CdTe/BiOI samples (*λ* excitation = 365 nm)

The photoluminescence emission spectroscopy (PL) is conducted to further study the transfer behavior of photogenerated charge carriers. As shown in Fig. 9, all samples show a broad PL emission peak at about 450–500 nm with the excitation at 365 nm. The bare TiO_2_(S) has a strong emission peak, while TiO_2_(S)/CdTe/BiOI sample displays lower intensity than that of TiO_2_(S). This phenomenon indicates that the recombination rate of photogenerated charge carriers was efficiently restrained after decorating CdTe and BiOI on the surface of TiO_2_. Furthermore, the TiO_2_(H) /CdTe /BiOI exhibits significantly diminished PL intensity in comparison with the other samples, which is cause by the faster transfer of electrons and holes from CdTe QDs or BiOI nanosheets to the surface of TiO_2_. The PL results are consistent with the result from photodegradation experiment.

**Fig. 10:**
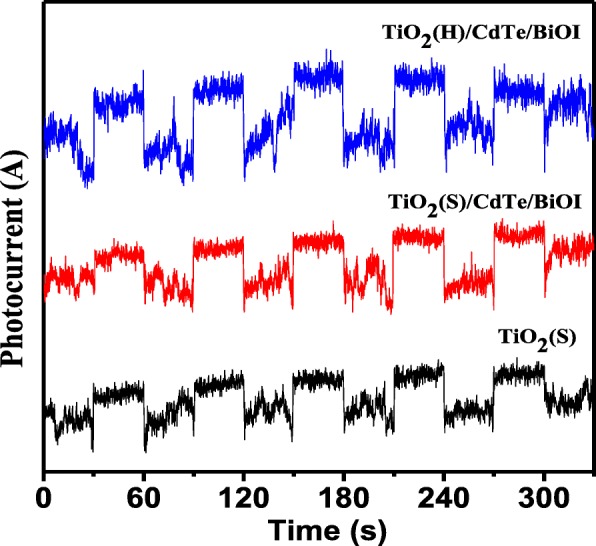
The transient photocurrent response of TiO_2_(S), TiO_2_(S)/CdTe/BiOI, and TiO_2_(H)/CdTe/BiOI

The photocurrent of samples is shown in Fig. 10. It was worth noting that the TiO_2_(S)/CdTe composites exhibited higher photocurrent response than that of pure TiO_2_(S) and TiO_2_(S)/CdTe/BiOI composites. Therefore, the increased photocurrent could be mainly attributed to the efficient photogenerated separation and migration, which benefits for the photocatalytic performance.

**Scheme 2:**
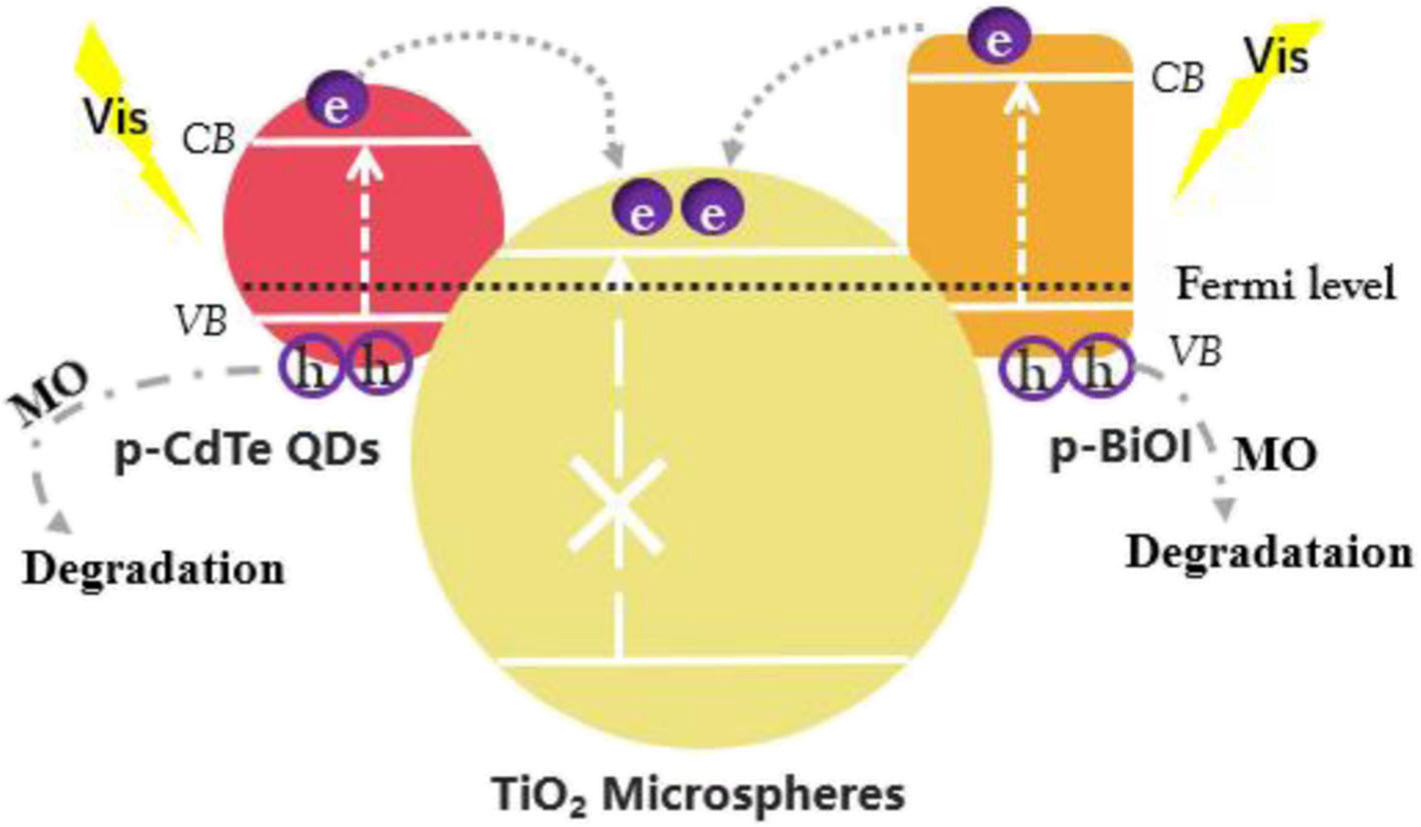
Illustration of photo-induced charge transfer in ternary TiO_2_/CdTe/BiOI photocatalytic system

Based on the results and discussions above, we propose a synergistic CdTe QDs/BiOI sensitization mechanism of the exciton transfer in TiO_2_/CdTe/BiOI to explain the enhanced photo activity, as illustrated in Scheme 2. It is well known that TiO_2_ with a wide bandgap (3.02 eV) could only utilize the UV region in solar light, while the narrow bandgap CdTe QDs (~ 1.5 eV) [32] and BiOI nanostructures (~ 1.8 eV) [33] can be excited by photons in the visible range. In addition, a p-n junction is formed between p-type BiOI and n-type TiO_2_ when Fermi levels reached equilibrium, which facilitate photo-induced electrons to migrate from CB of BiOI to that of TiO_2_ [17, 34]. Similarly, a type II heterojunction is formed between p-type CdTe [18] and TiO_2_ microspheres; thus, electrons in the CB of CdTe QDs can transfer to TiO_2_ [35]. Therefore, the lifetime of the photogenerated electron and hole is prolonged, which is beneficial for the degradation towards MO.

## Conclusions

In summary, a series of TiO_2_-based photocatalysts were synthesized by a facile hydrothermal method. Modifications by BiOI and CdTe QDs were carried out to fabricate binary and ternary heterostructures, and the narrow bandgap semiconductors extended light response for the hybrid photocatalysts. In the case of ternary TiO_2_/CdTe/BiOI heterostructured photocatalyst, the BiOI flakes and CdTe QDs act as sensitizers on one hand, which are excited by simulated solar light and transfer electrons to TiO_2_. Meanwhile, the TiO_2_ microspheres serve as separation centers for the photo-induced charges on the other hand; thus, the synergistic effect among TiO_2_, CdTe, and BiOI enhances the photocatalytic removal of MO. In addition, hollow TiO_2_ precursors were also employed to fabricate TiO_2_/CdTe/BiOI heterostructures, and the improved photocatalytic performance towards MO degradation is attributed to a higher surface area and dispersion of BiOI components. The strategy of material regulation and incorporation will provide possibilities for the design of the multi-component semiconductor photocatalysts.

## Abbreviations

TiO_2_(H): TiO_2_ hollow microspheres
TiO_2_(S): TiO_2_ solid microspheres
TiO_2_/BiOI: TiO_2_ spheres modified with BiOI
TiO_2_/CdTe: TiO_2_ spheres modified with CdTe QDs
TiO_2_/CdTe/BiOI: TiO_2_, CdTe, and BiOI ternary composites
